# Corrosion Fatigue Degradation Characteristics of Galvanized and Galfan High-Strength Steel Wire

**DOI:** 10.3390/ma16020708

**Published:** 2023-01-11

**Authors:** Yue Zhao, Botong Su, Xiaobo Fan, Yangguang Yuan, Yiyun Zhu

**Affiliations:** 1 School of Civil Engineering and Architecture, Xi’an University of Technology, Xi’an 710048, China; 2 Xi’an Municipal Engineering Design & Research Institute Co., Ltd., Xi’an 710068, China; 3 School of Architecture and Civil Engineering, Xi’an University of Science and Technology, Xi’an 710054, China

**Keywords:** high strength steel wire, corrosion fatigue, uniform corrosion, pitting corrosion, traffic load

## Abstract

Cables are the main load-bearing components of a cable bridge and typically composed of high strength steel wires with a galvanized coating or Galfan coating. Galfan steel wire has recently started to be widely used because of its better corrosion resistance than galvanized steel wire. The corrosion characteristics of the coating and the difference in the corrosion fatigue process of the two types of steel wire are unclear. To further improve the service performance and maintenance of cable bridges, this study investigated the corrosion characteristics of galvanized steel wire and Galfan steel wire through accelerated corrosion tests and established a time-varying model of uniform corrosion and pitting corrosion of high-strength steel wire. Then, a long-span suspension bridge was taken as the research object, and the corrosion fatigue degradation of the two kinds of steel wire under a traffic load was analyzed on the basis of traffic monitoring data. The results showed that the uniform corrosion of the two types of steel wire conformed to an exponential development trend, the corrosion coefficient of galvanized steel wire conformed to the normal distribution, and the corrosion coefficient of Galfan steel wire conformed to the Cauchy distribution. The maximum pitting coefficient distribution of the two kinds of steel wire conformed to the generalized extreme value distribution. The location parameters and scale parameters of the two distributions showed an exponential downward trend with the increase of corrosion duration. When the traffic intensity was low, the corrosion characteristics of the steel wire was the main factor affecting its service life, and the average service life of Galfan steel wire was significantly higher than that of galvanized steel wire. Under a dense traffic flow, the service life of the steel wire was mainly controlled by the traffic load, and the service life of Galfan steel wire was slightly improved. Effective anti-corrosion measures are a key factor for improving the service life of steel wire.

## 1. Introduction

High-strength steel wire is the key load-bearing component of cable bearing bridges, such as cable-stayed bridges and suspension bridges, and will degrade during operation [1]. The reliability of a bridge in operation is deeply influenced by the corrosion degradation of its components [2]. The corrosion problem of cable systems has aroused extensive consideration from scholars. The main types of cable structure are parallel wire rope and steel strand; both are composed of single steel wire. The earliest cable steel wires were all-steel wires without coating. The cable was wrapped with a protective sleeve outside and filled with barrier materials inside, but the anti-corrosion effect was poor, as proven in practice. To resist the corrosion of environmental factors, the cable components began to have a coating on the surface of the high-strength steel wire, to isolate the corrosion medium. Early cable bearing bridges mostly used galvanized high-strength steel wire, and relevant research has mainly focused on galvanized steel wire. The corrosion development law of galvanized coating is relatively clear. In recent years, Galfan coating steel wire with better corrosion resistance has gradually started to be widely used. Its corrosion resistance is better than that of galvanized steel wire, but the corrosion characteristics of this coating lack systematic research. The deterioration of the steel wire is the result of the combined effect of corrosion and fatigue. The corrosion characteristics of a steel wire coating directly affect the corrosion state of the steel substrate and the subsequent fatigue crack growth. The difference of corrosion fatigue properties between the two types of steel wires is unclear. To further improve the service performance and operation and maintenance level of a cable load-bearing structure, this work intended to conduct an experimental study on the corrosion characteristics of galvanized steel wire and Galfan steel wire, analyze the degradation characteristics of the two types of high-strength steel wire, and discuss the influence of corrosion and fatigue on the degradation of high-strength steel wire.

The degradation of high-strength steel wire is the result of the combined effect of corrosion and fatigue. Simple corrosion and fatigue degradation of steel wire is relatively slow. The pitting corrosion pits produced along with the uniform corrosion of steel wire provide conditions for the initiation of fatigue cracks, thus greatly reducing the fatigue life of steel wire; the fatigue strength of the steel wire decreases with the increase of corrosion [3,4]. Betti et al. conducted in-depth research on the deterioration mechanism of the high-strength steel wire of a suspension bridge, studied the corrosion evolution of galvanized and non-galvanized steel wire under different environmental conditions through an accelerated cyclic corrosion test, and pointed out that the uneven change of the steel wire section along the length reduced the elongation of the steel wire [5]. Nakamura, Suzumura, and others researched the influence of reagent concentration, ambient temperature, and humidity on the corrosion rate of galvanized steel wire through experiments, given the loss rate of the galvanized layer of galvanized steel wire. They pointed out that the main reason for the deterioration of the properties of corroded steel wire is the reduction of elongation, torsional strength, and fatigue strength [6,7,8,9]. Lan et al. conducted an acid salt spray test and fatigue test on high-strength steel wire and fitted the corrosion fatigue life of steel wire based on the Weibull distribution. The change trend of the fatigue life of steel wire and cable components with the development of the corrosion process is basically consistent, and the fatigue life of a stay cable decreases significantly as the corrosion degree of the steel wire increases [10]. The above research confirmed that the fatigue life of steel wire decreases due to corrosion, from practical engineering and laboratory research. Jiang et al. and Wang et al. used solutions to create corrosive environments and studied the effects of different solutions, solution concentrations, stress amplitudes, and load frequencies on fatigue life. The corrosion fatigue performance of steel wire in acidic environments was the worst, and electrochemical reaction greatly reduced the life of the steel wire [11,12]. Sun established a corrosion fatigue degradation model of steel wire based on fracture mechanics and compared it with test results, which proved that the proposed model could better simulate the evolution of corrosion fatigue of steel wire [13]. Li et al. established improved uniform corrosion and pitting models for high-strength steel wire, verified that the maximum pitting factor obeys a Gumbel distribution based on an accelerated corrosion test, fitted relevant parameters, and studied corrosion fatigue through finite element simulation [14]. Jiang et al. measured the corroded steel wire 3D profile and proposed that the pitting depth of steel wire follows a normal distribution and that the location and scale parameters increase with the degree of corrosion. A method for predicting the residual life of corroded steel wire based on 3D measurement and AFGROW software was established [15]. The basic process and principles of corrosion fatigue degradation have been confirmed by scholars.

The above research mainly focused on the corrosion characteristics of galvanized steel wire. Compared with galvanized steel wire, galvanized aluminum high-strength steel wire has been gradually applied to engineering construction in recent years. However, the research on its corrosion behavior characteristics is relatively scarce. Xue et al. [16] studied the corrosion fatigue behavior of Galfan coating, and Cao et al. [17] studied the effect of Nd on the corrosion behavior of Zn-5Al (wt.%) alloy in neutral 3.5wt.%NaCl solution using electrochemical impedance spectroscopy. The addition of Nd can improve the corrosion resistance of Zn-5Al alloy. The above studies are important references for the study of Galfan coating corrosion resistance performance. Nonetheless, the development of Galfan coating corrosion resistance and pitting has not been systematically studied, and the difference in the service life between the two kinds of steel wires is unclear.

Systematic studies on the corrosion resistance of Galfan wire coating and the difference between its corrosion fatigue and the fatigue of galvanized wire are few. The effect on improving the service performance of cable structures in engineering applications is unclear. In this study, the corrosion resistance of galvanized and Galfan high-strength steel wires was studied using an accelerated corrosion test. On the basis of the test results, a uniform corrosion development model of the two kinds of steel wires was established, and a dynamic distribution model of the maximum pitting coefficient was established using Gumbel distribution. The time-varying characteristics of the scale and location parameters of the maximum pitting coefficient distribution for the two types of steel wire are given. On the basis of the traffic load monitoring data of a bridge during operation, the corrosion fatigue degradation characteristics of the steel wires of the cable components in the service period were analyzed, which can provide a reference for the design and maintenance of the components of bridge structure cables.

## 2. Accelerated Corrosion Experiment

Corrosion tests can generally be divided into two categories: one is the traditional corrosion tests under natural conditions. The real corrosion conditions of test objects can be obtained by directly exposing the test samples to the real environment, but the time cost is high and the test cycle is too long. The other is accelerated corrosion tests under a laboratory environment. By putting the test samples into a corrosion chamber and using a salt spray environment, atmospheric pressure, temperature, and other factors to accelerate corrosion, the test time is greatly shortened. With the rapid development of bridge component materials and the increasing demand for corrosion-resistance research, the salt spray test has become the most commonly used method for cable corrosion research. Based on the specification “Corrosion Test in Artificial Atmospheres—Salt Spray Test”(GB/T 10125-2012) [18], a neutral salt spray test was selected to study the corrosion characteristics of galvanized and Galfan high-strength steel wire. The corrosion atmosphere was formed using a salt spray test chamber, and an accelerated corrosion effect was achieved by combining temperature and air pressure. Given the few parameters of the accelerated corrosion test, the concentration and continuity of the salt spray, temperature, and pressure in the test chamber were mainly guaranteed during the test.

The accelerated corrosion time and number of test pieces are presented in Table 1. With reference to existing research results, the planned test duration for galvanized steel wire was 510 h, and the test duration for Galfan steel wire was 1445 h. Each group of galvanized steel wires had 5 test pieces, and the first 10 groups of Galfan steel wire had 5 steel wires. To ensure the accuracy of data, each group of Galfan steel wires had 10 steel wires. Before placing the steel wire test pieces, all steel wires were weighed and numbered, and then the test pieces were placed in a salt spray test chamber for artificial atmospheric corrosion. The scheduled steel wire test pieces were taken out in batches, according to the planned corrosion time period, and then the corrosion products were removed using a combined chemical method and physical method, according to the corrosion product removal specification “Corrosion of Metals and Alloys–Removal of Corrosion Products from Corrosion Test Specimens” (GB/T 16545) [19]. The pickling solution was a saturated solution of NH_3_CH_2_COOH, where 1000 mL solution was prepared by mixing 250 g NH_3_CH_2_COOH and distilled water. The specific steps were as follows: (1) Put the sample into the saturated NH_3_CH_2_COOH solution (pickling solution) at 20~25 °C and soak it for 10 min. (2) Rub off the residual corrosion products with abrasive paper. (3) Successively put it into water and alcohol 5 times for cleaning. (4) Wipe it with a towel and dry it after cleaning. After each group of steel wire samples was taken out at specific times, the corrosion products were removed and weighed according to the above steps.

### 2.1. Specimens of High-Strength Steel Wire

The high-strength steel wire used in the test was provided by a cable manufacturer. The material parameters of the steel wire samples were tested, and the results are given in Table 2. According to the mass and density of the coating, the thickness of the zinc coating was 28.05μm, and the thickness of the Galfan coating was 29.53 μm. Galvanized steel wire coatings consisted of pure zinc, and Galfan steel wire consisted of 5% aluminum zinc alloy and a small amount of mixed rare earth elements. The chemical composition of the steel wire coating is shown in Table 3.

The steel wire was cut to make an experimental sample, and the sample length was about 20 cm. Before the test, all steel wire samples were cleaned, dried, numbered, measured, and weighed one by one. After the preparation, they were put into the salt spray box.

### 2.2. Test Device and Accelerated Corrosion Medium

The test materials required for the neutral salt spray test included: steel wire sample, salt spray box, electronic balance, C_2_H_5_OH, NaCl, and pickling agent. The quality of the steel wire was measured using a high-precision electronic balance. The reagent grade used in the test was chemically pure. The parameters of the relevant instruments and chemical reagents are provided in Table 4.

**Figure 1:**
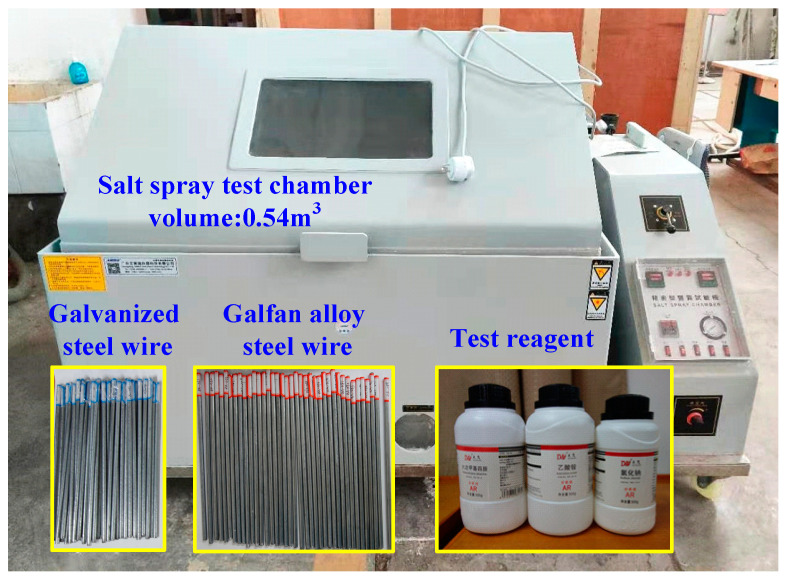
Equipment and materials for the salt spray test device.

A Zhongte LX120 multi-functional climate and environment salt fog test chamber was adopted for the test salt fog chamber, which can realize the simulation of a salt fog environment, high temperature, and high pressure conditions, as well as the coupled effect of different conditions. The technical parameters are listed in Table 5. The experimental equipment and materials are shown in Figure 1.

**Figure 2:**
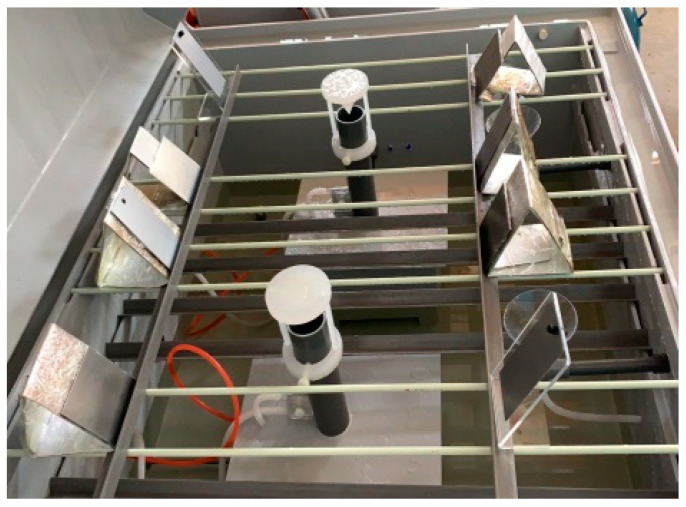
Steel reference test.

According to the requirements of the specification for the neutral salt spray test and to test the reproducibility of the test equipment results, a steel reference test verification was carried out. Four defect-free CR4 grade cold-rolled carbon steel plates with a thickness of 1 mm were selected. After cleaning, the back of the samples was protected with a film, and a sample was placed at the four corners of the salt spray box for 48 h, as shown in Figure 2. The specific test parameters were set according to the specifications. The volume of the salt spray box was 0.6 m^3^. No solution accumulated on the top of the box on the sample, and the spray was always uniform. The pH value of the spray solution collected by the collector was in the range 6.5–7.2. The test temperature was 35 ± 1 °C, and the concentration of NaCl was 50 g/L. The settling rate of the salt spray met the specifications.

The reference sample was taken out immediately after the test, and ammonium acetate solution and a mechanical cleaning method were used to remove the corrosion products. The mass loss per unit area obtained after weighing is presented in Table 6. The loss of each test piece was within the range of 70 ± 20 g/m^2^ required by the specification, indicating that the equipment operated normally and met the operational requirements of the neutral salt spray test. The steel wire corrosion tests were then carried out according to the specification requirements. The test solution was NaCl solution with a concentration of 50 g/L ± 5 g/L, the pH value of spray solution was 6.5 to 7.2, and the temperature was 35 ± 2 °C.

## 3. Corrosion Phenomenon

**Figure 3:**
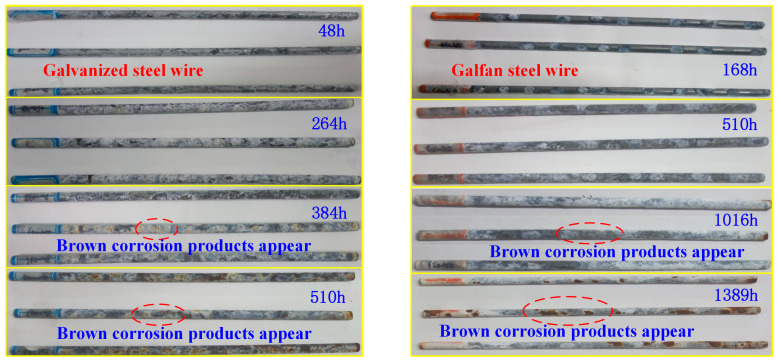
Corrosion morphology in the different stages of corrosion.

The box was opened, and the corresponding test pieces were taken out, according to the time specified in the test plan. They were cleaned and dried in strict accordance with the corrosion product removal specification. Figure 3 shows the corrosion morphology of a single Galfan steel wire at different corrosion stages. The degree of corrosion of the steel wire gradually increased with time, and the corrosion resistance of the Galfan steel wire was much better than that of the galvanized steel wire. The early corrosion of the steel wire was reflected in the loss of luster of the coating, accumulation of salt on the surface of the steel wire, and the gradual production of uniformly distributed white accumulation products on the surface of the steel wire.

The galvanized steel wire and the Galfan steel wire were completely covered by white corrosion products from the 264 h and 510 h, respectively. After the corrosion products were removed, part of the coating had lost luster, indicating that the coating had been corroded at this time and part of the iron matrix had begun to be exposed. At 384 h and 1016 h, some corrosion spots had appeared on the surface of the steel wire, indicating that the iron matrix under the partial coating of the steel wire had started to corrode at this time, producing reddish brown corrosion products. At 510 h, many reddish brown corrosion products had appeared at the middle and end of the galvanized steel wire, and the reddish brown corrosion products had connected into sheets. At 1389 h, the same situation had occurred to the Galfan steel wire. At this time, uneven pits had appeared on the steel wire surface after the corrosion products had been removed. The corrosion of steel wire can be generally divided into two parts: The first part is the corrosion of the surface coating. When the corrosion depth exceeds the coating thickness, the corrosion of the second part of the steel wire matrix begins. Owing to the protection of oxidation products formed after the coating corrosion, the corrosion rate of the steel wire matrix decreases.

## 4. Corrosion Process

### 4.1. Uniform Corrosion

The development of the uniform corrosion of steel wire is mainly affected by two factors: the corrosion time, and the uniform corrosion rate. As the uniform corrosion depth is not easy to obtain directly, it is generally described using the volumetric method, weight-loss method, or other methods. In this study, the weight-loss method was used to describe the uniform corrosion of steel wire, which is given as Equation (1). With reference to the specification for removal of corrosion products, the chemical substances generated after steel wire corrosion can be removed without damaging the metal matrix, and the quality loss of metal in the corrosive environment can be accurately measured, to evaluate the degree of corrosion of steel wire.

(1) $$\psi =\frac{m_{0}/l_{0}-m_{1}/l_{1}}{m_{0}/l_{0}}\times 100\%$$

where *ψ* represents the loss rate of steel wire mass, *m*_0_ represents the quality of steel wire before corrosion, *l*_0_ represents the length of steel wire before corrosion, *m*_1_ represents the quality of steel wire after corrosion, and *l*_1_ represents the length of steel wire after corrosion.

Owing to the large slenderness ratio of the steel wire specimen, the sectional area of both ends of the steel wire is small and the calculation constant is large, so the length change caused by corrosion can be ignored. That is $l_{0}=l_{1}$. Therefore, Equation (1) can be rewritten as Equation (2):

(2) $$\psi =\frac{m_{0}-m_{1}}{m_{0}}$$

According to Equation (3), the mass loss of steel wire is converted into the coating and corrosion depth of steel wire *du*.

(3) $$du=\frac{\psi }{A\rho }=\frac{m_{0}-m_{1}}{\pi D\rho l_{0}}$$

where *A* is the surface area of steel wire, $A=\pi Dl_{0}$, *D* is the initial diameter of steel wire, *ρ* is the material density, and $l_{0}$ is the length of steel wire.

**Figure 4:**
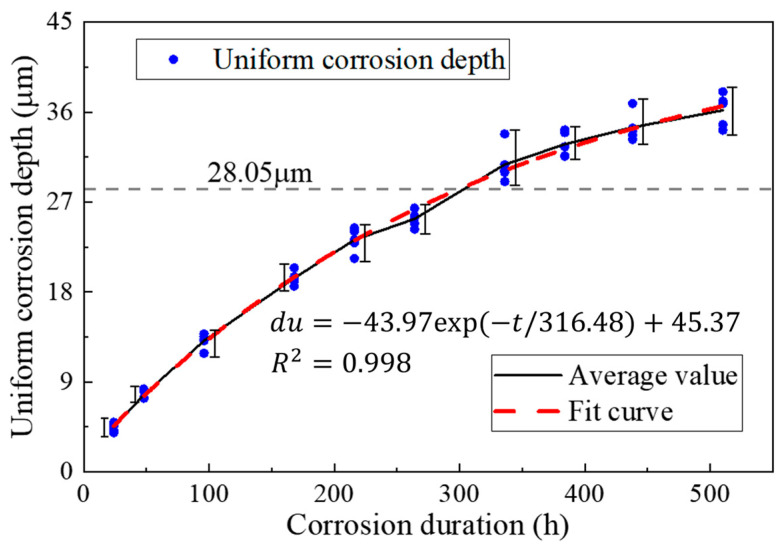
Average mass loss per unit length of galvanized steel wire.

**Figure 5:**
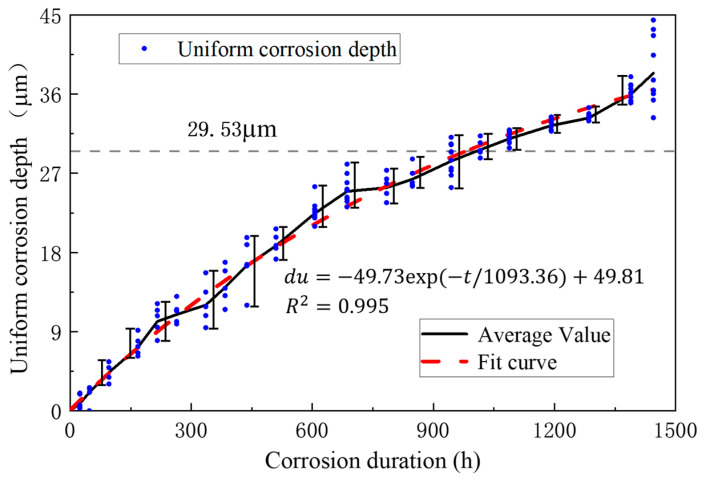
Average mass loss per unit length of Galfan steel wire.

Figure 4 and Figure 5 show the average mass loss per unit length of all steel wire samples, indicating that the overall distribution of test results was relatively regular. The change of steel wire mass loss at each stage was stable, without obvious mutations, indicating that the test effect was good. The mass loss of Galfan steel wire increased obviously with the increase of corrosion time. During the entire corrosion period, the increasing trend of corrosion quality was close to an exponential change, and the corrosion rate gradually slowed down.

**Figure 6:**
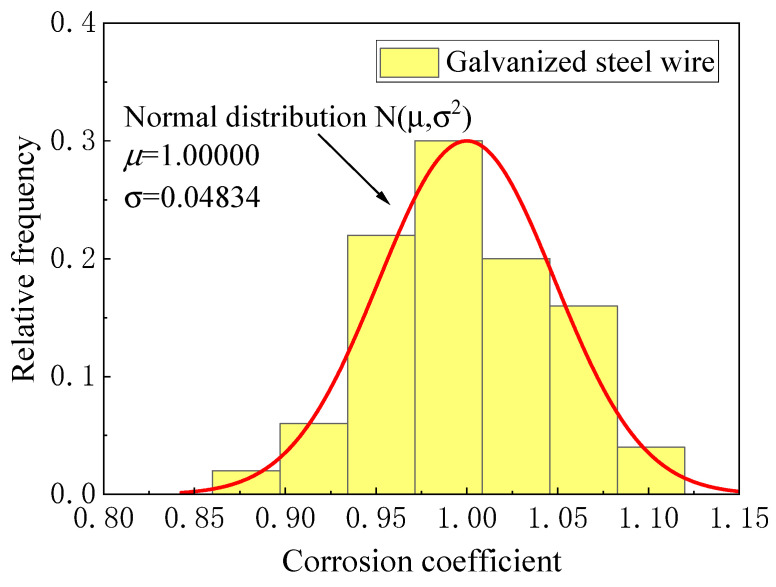
Corrosion coefficient distribution of galvanized steel wire.

**Figure 7:**
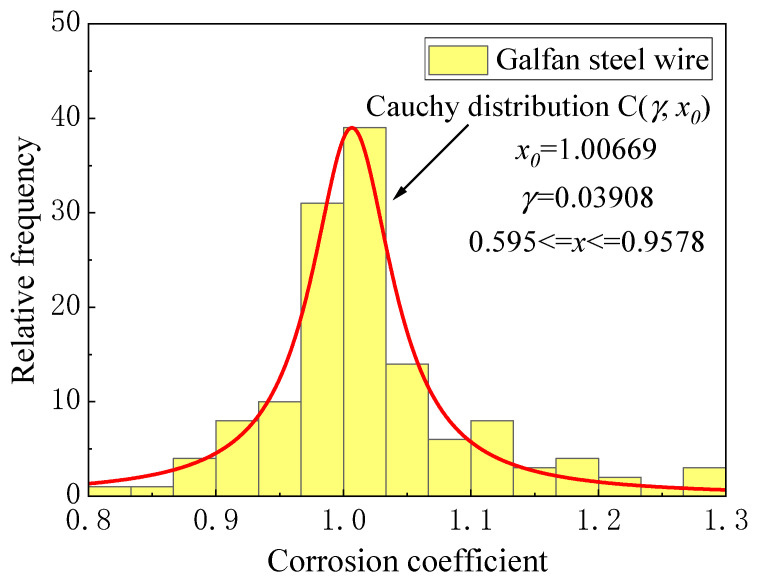
Corrosion coefficient distribution of Galfan steel wire.

Owing to the influence of the coating thickness and corrosion randomness, the corrosion rate of different steel wires varies, so the steel wire corrosion coefficient was introduced to describe the randomness. Figure 6 and Figure 7 show the fit distribution law of the two kinds of steel wires. The corrosion coefficient of the galvanized steel wire conformed to the normal distribution N(*μ*, *σ*^2^) with *μ* = 1.00 and *σ* = 0.0483, whereas the Galfan steel wire conformed to the Cauchy distribution C(*γ*, *x*_0_) with *x*_0_ = 1.00 and *γ* = 0.0391.

Therefore, the uniform corrosion rate of the two kinds of steel wire can be expressed as in Equation (4).

(4) $$\{\begin{array}{c} du_{Zn}\left(t\right)=\psi_{Zn}*\left(-43.97*exp\left(-t/316.48\right)+45.37\right)\;\;\;\;\;\;\;\;\;\;\;\;\;\;Galvanized\;steel\;wire\\ du_{Gal}\left(t\right)=\psi_{Gal}*\left(-49.73*exp\left(-t/1093.36\right)+49.81\right)\;\;\;\;\;\;\;\;\;\;\;\;\;\;\;\;\;\;Galfan\;steel\;wire \end{array}$$

where *t* is the corrosion duration, $\psi_{Zn}$ conforms to the normal distribution N(*μ*, *σ^2^*) with *μ* = 1.00, *σ* = 0.04834, and $\psi_{Gal}$ conforms to the Cauchy distribution C(*γ*, *x*_0_) with *x*_0_ = 1.00669, *γ* = 0.03908.

### 4.2. Pitting Corrosion

The key factor causing cracks in corrosion fatigue is pitting corrosion, which is accompanied by uniform corrosion. When a passivation or film forms on the metal material surface, a small and deep corrosion pit is generated on the substrate surface after the protective layer is consumed. Owing to the stress concentration effect, the stress at the edge of the corrosion pit is far greater than the overall stress level. When the depth of the corrosion pit increases to a certain extent, it becomes a crack. A crack usually occurs at the position with the greatest pitting corrosion, so the deepest pitting corrosion determines the working state of the steel wire and is a key analysis point in corrosion fatigue analysis.

**Figure 8:**
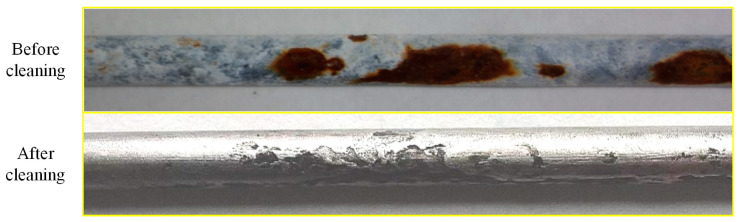
Surface morphology of steel wire.

Figure 8 shows the surface morphology of the steel wire after cleaning. The pits on the surface of the steel wire are obvious. To further determine the distribution characteristics of the pitting corrosion of the different types of steel wire, the steel wire indication was detected using a 3D shape scanner, and a 3D model of the steel wire surface was established based on the regression of 2D scanning results. As the steel wire specimen was not completely straight, the surface profile presented an irregular curve shape as a whole. To eliminate the influence of the steel wire specimen’s own curved shape on the test results, the small window moving average automatic baseline correction method was used to estimate the baseline corresponding to the measured profile. According to the difference between the measured contour coordinates and the baseline coordinates, the pitting depth on the axial length of the steel wire could be determined.

**Figure 9:**
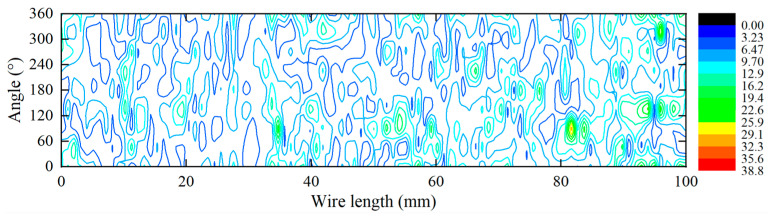
Regression of the 3D corrosion morphology of galvanized steel wire.

**Figure 10:**
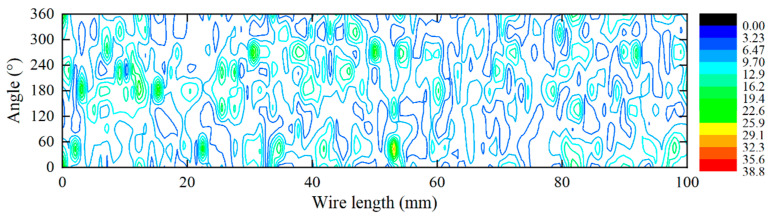
Regression of the 3D corrosion morphology of Galfan steel wire.

The pitting depth could be calculated using the uniform corrosion depth and maximum pitting factor [20]. Figure 9 and Figure 10 show the 3D surface regression profile of the steel wire surface. According to the 40 measured surface profiles and regression analysis results, the block maximum value method was used to obtain a sample of the maximum pitting factor in each exposure period. The analysis accuracy of the block maximum method is directly affected by the selected block size, and the sample size of the block maximum should be sufficiently large. After comprehensive consideration, this study determined that the block size for calculating the pitting factor was 10 mm. The maximum pitting depth was taken every 10 mm along the length of the steel wire, and the maximum pitting factor was calculated according to Equation (5).

(5) G(t)=da(t)/du(t)

where *G*(*t*) is the maximum pitting factor, *da*(*t*) is the maximum pitting depth, and *du*(*t*) is the uniform corrosion depth in the same period.

**Figure 11:**
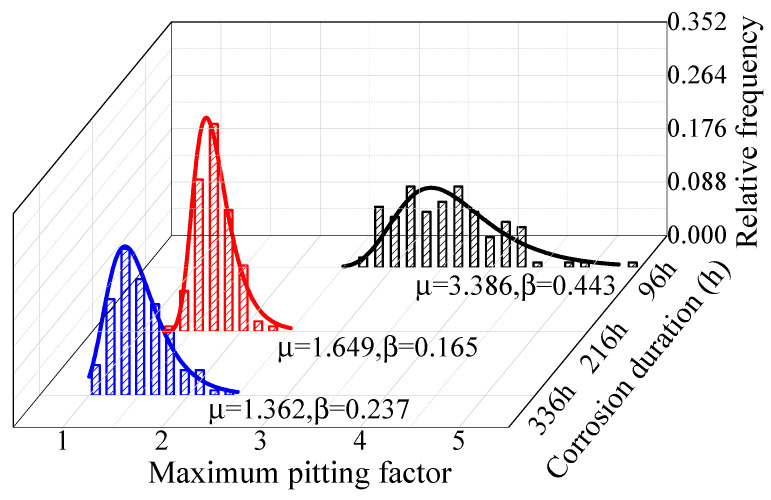
Fitting results of maximum pitting factor of galvanized steel wire.

**Figure 12:**
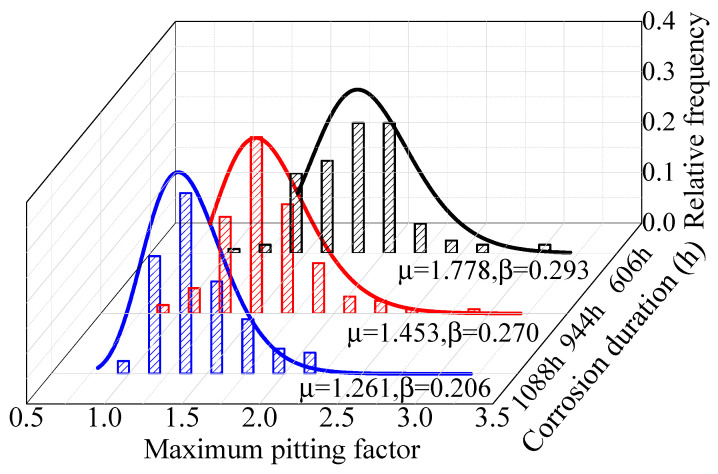
Fitting results of maximum pitting factor of Galfan steel wire.

The existing research ignored the time-varying characteristics of the maximum pitting factor and posited that the maximum pitting factors in different periods have the same distribution characteristics. Figure 11 and Figure 12 show the fitting results of the maximum pitting factor of steel wire in certain periods. The maximum pitting factor has obvious time-varying characteristics. Gumbel distribution was used to fit the maximum pitting factor. The relationship between the pitting system and location parameters, scale parameters, and corrosion time can be expressed as in Equation (6).

(6) $$Z_{p}=exp\left\{-exp\;\left[-\left(\frac{x-\mu \left(t\right)}{\sigma \left(t\right)}\right)\right]\right\}$$

where μ(t) and σ(t) are the location parameters and scale parameters corresponding to the accelerated corrosion duration *t*, respectively.

For galvanized steel wire, the pitting factor was larger at the early stage of corrosion; μ was 3.386 at 96 h. The pitting factor decreased significantly with the increase of time at the later stages; μ was 1.649 at 216 h. The change amplitude of Galfan steel wire was smaller than that of the galvanized steel wire but also decreased with the increase of time; μ was 1.778 at 606 h and 1.261 at 1088 h. The increase of the pitting depth was limited, and estimating the corrosion process of the degraded steel wire according to a single distribution law may underestimate the service state of steel wire. Furthermore, the fitting analysis of the location parameters and scale parameters of the pitting distribution function in different corrosion stages showed that the distribution parameters of the two coatings conformed to the exponential change law, which could be calculated according to Equations (7) and (8).

(7) $$\{\begin{array}{c} \mu_{Zn}\left(t\right)=9.8698\exp\left(-t/59.2058\right)+1.3595\\ \sigma_{Zn}\left(t\right)=1.3967\exp\left(-t/58.0061\right)+0.2190 \end{array}\;\;\;\;\;\;\;\;\;\;\;\;\;\;\;\;\;Galvanized\;steel\;wire$$

(8) $$\{\begin{array}{c} \mu_{Gal}\left(t\right)=18.7549\;exp\left(-t/71.0266\right)+1.7746\\ \sigma_{Gal}\left(t\right)=4.5949\;exp\left(-t/55.5349\right)+0.3375\;\;\; \end{array}\;\;\;\;\;\;\;\;\;\;\;\;\;\;\;\;\;\;\;\;\;\;Galfan\;steel\;wire$$

where $\mu_{Zn}\left(t\right)$, $\sigma_{Zn}\left(t\right)$, $\mu_{Gal}\left(t\right)$, and $\sigma_{Gal}\left(t\right)$, respectively, represent the location parameters and scale parameters of the maximum pitting coefficient distribution between the zinc coating and Galfan coating corresponding to the accelerated corrosion duration *t*.

## 5. Corrosion Fatigue of High-Strength Steel Wire

### 5.1. Corrosion Fatigue Degradation Model

When the protection system of cable components is damaged because the air contains water, salt, and other substances, the corrosion factors of the external environment enter the cable body and cause steel wire corrosion. The corrosion of steel wire generally goes through the following three stages [21]: (1) uniform corrosion and pitting corrosion, (2) pitting crack development, and (3) corrosion fatigue crack growth. Uniform corrosion refers to uniform corrosion and detachment of the steel wire surface, which belongs to pure chemical reaction and directly causes the reduction of the steel wire diameter. The reduction of diameter is approximately equal along the length of steel wire. Owing to the non-uniformity of the material, the diameter of the steel wire decreases, accompanied by pitting corrosion randomly distributed on the surface of the steel wire. Uniform corrosion includes the corrosion consumption of the steel wire coating and the time needed for uniform corrosion and pitting of the steel wire matrix. The specific development rules of uniform corrosion and pitting were obtained through accelerated corrosion tests in this study.

On the basis of the unit area *A*_0_ of pitting calculation, Equation (9) can be used to obtain the maximum pitting coefficient distribution of *A_k_* in any area [22,23]. When estimating the maximum pitting coefficient per unit area, Saint Venant’s principle must be considered. The minimum length of the analysis unit should be greater than twice the diameter of the steel wire.

(9) $$\mu_{k}=\mu_{0}+\frac{1}{\sigma_{0}}ln(\frac{A_{k}}{A_{0}}),\sigma_{k}=\sigma_{0}$$

where $A_{k}$ is the surface area of the analysis target, and $A_{0}$ is the surface area of unit area. $\mu_{0}$ and $\sigma_{0}$ are the location parameters and scale parameters of the corresponding fitting analysis results by exponential function.

Y. Kondo pointed out that the initiation of fatigue cracks is caused by pitting, and the transition from pitting depth to fatigue cracks is related to the stress amplitude of the steel wire. When the stress amplitude is large, the cracks easily occur in smaller pits, and vice versa. The development of pitting pits into fatigue cracks can be described based on the theory of fracture mechanics. The stress intensity factor *K* is introduced to describe the strength of the stress field near the crack tip. The generation of cracks is mainly affected by the alternating stress field at this location. The amplitude of the stress intensity factor ΔK can be calculated according to Equation (10).

(10) $$\Delta K=F_{a}\left(\frac{a}{b}\right)\Delta \sigma_{a}\sqrt{\pi a}+F_{b}\left(\frac{a}{b}\right)\Delta \sigma_{b}\sqrt{\pi a}$$

where *a* is the crack depth, *b* is the diameter of steel wire, $\Delta \sigma_{a}$ is the equivalent axial stress amplitude, and $\Delta \sigma_{b}$ is the equivalent axial stress amplitude. $F\left(\frac{a}{b}\right)$ is calculated using Equation (11) [24].

(11) $$\{\begin{array}{c} F_{a}\left(\frac{a}{b}\right)=0.92\cdot \frac{2}{\pi }\cdot \sqrt{\frac{2b}{\pi a}\cdot tan\frac{\pi a}{2b}}\cdot \frac{0.752+1.286\left(\frac{a}{b}\right)+0.37{\left(1-sin\frac{\pi a}{2b}\right)}^{3}}{cos\frac{\pi a}{2b}}\\ F_{b}\left(\frac{a}{b}\right)=0.92\cdot \frac{2}{\pi }\cdot \sqrt{\frac{2b}{\pi a}\cdot tan\frac{\pi a}{2b}}\cdot \frac{0.923+0.199{\left(1-sin\frac{\pi a}{2b}\right)}^{4}}{cos\frac{\pi a}{2b}} \end{array}$$

where $F_{a}\left(\frac{a}{b}\right)$ denotes axial stress, $F_{b}\left(\frac{a}{b}\right)$ denotes bending stress, *a* is the crack depth, and *b* is the diameter of steel wire.

The corrosion cracking of steel wire is generated from pitting to cracks. When the stress intensity factor at the pitting reaches the threshold value for crack growth of 2.8 MPa·m^1/2^ [25], the pitting development is transformed into the crack development stage. The Paris formula is the most widely used method for the growth rate analysis of metal corrosion fatigue cracking, and the crack growth rate is expressed as the fatigue crack growth rate [26].

(12) $$\frac{da}{dt}=C{\left(\Delta K\right)}^{m}N$$

where ΔK is the effective stress intensity factor amplitude, and *C* and *m* are the parameters of the Paris criterion.

To comprehensively consider the impact of the daily traffic flow intensity level on the crack development rate, a crack depth development model was established based on the proportion of daily traffic flow operations, as given in Equation (13).

(13) $$\{\begin{array}{l} a_{i}=\Delta a+a_{i-1}\;\;\;\;\;\;\;\;\;\;\;\;\;\;\;\;\;\;\;\;\;\;\;\;\;\;\;\;\;\;\;\;\;\;\\ \Delta a=C\sum n_{q}\;[\sum e_{j}{\left(\Delta K_{qj}\right)}^{m}N_{qj}] \end{array}$$

where $a_{i}$ is the depth of the crack at time *i*, Δa is the increment of the crack, $e_{j}$ is the operating time of traffic flow with different intensities, $\sum e_{j}=24$ h, and $\Delta K_{j}$ and $N_{j}$ are the stress intensity factor range and the number of cycles.

Mayrbaurl pointed out that the critical relative crack depth conforms to the logarithmic normal distribution, with an average value of 0.390 and a coefficient of variation of 0.414. Based on the tests, the maximum critical relative depth was 0.5, which can be used as the judgment standard for steel wire failure.

### 5.2. Protype Bridge and Traffic Load

**Figure 13:**
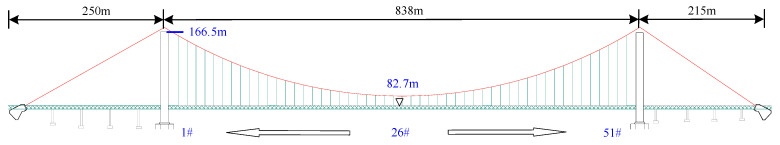
Elevation Layout of the Bridge.

**Figure 14:**
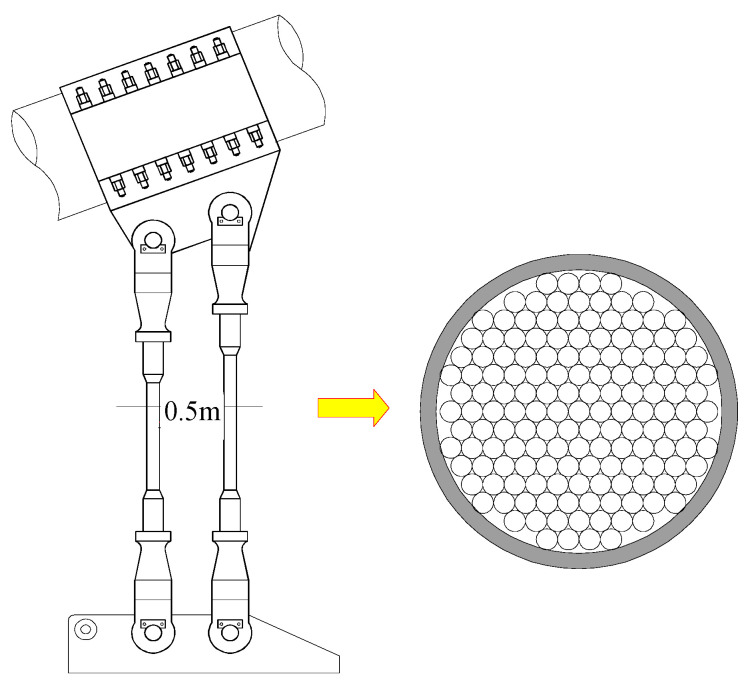
Schematic Diagram of a Suspender.

**Figure 15:**
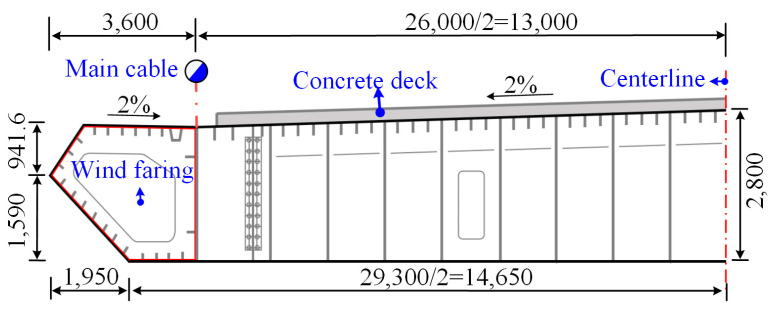
Section of the Main Girder.

To further analyze the corrosion fatigue degradation of the two types of high-strength steel wire under a load, this study took a long-span suspension bridge as the research background and took high-strength steel wire suspenders as the research object, to investigate the corrosion fatigue degradation of galvanized and Galfan high-strength steel wire under the same conditions. The main bridge structure of the bridge is a single span 838 m steel concrete composite girder suspension bridge. The main cable span is (250 + 838 + 215) m. The vertical layout is shown in Figure 13. Steel concrete composite girders are used as stiffening girders, and the steel beams are combined with concrete bridge decks through shear studs. The section layout is shown in Figure 14 and Figure 15. The full width of the stiffening girder is 33.2 m. The steel longitudinal beams on both sides are connected by steel cross beams. The center height is 2.8 m. The center distance between the webs of the longitudinal beams on both sides is 26.0 m. The bridge deck is a reinforced concrete bridge deck with a full width of 25.0 m and a thickness of 0.22 m. The standard spacing of suspender lifting points is 16 m. There are two suspenders for each lifting point, with 204 suspenders in total for the whole bridge, and 151 *ϕ*5 mm high-strength steel wires included in each suspender.

Stochastic traffic flow simulation is the mainstream method for long-span bridge operation evaluation. After the degradation of the suspender steel wire changes from pitting to cracking, it enters the main stage of crack development. The crack growth speed is mainly affected by the stress response of the suspender under the traffic load. Based on the measured traffic flow in a certain area, obtained by the traffic load monitoring system, this study analyzed and selected the data of representative periods, to simulate the formation of traffic flow for loading, so as to obtain the corrosion fatigue degradation of suspender steel wire under different levels of traffic loading.

**Figure 16:**
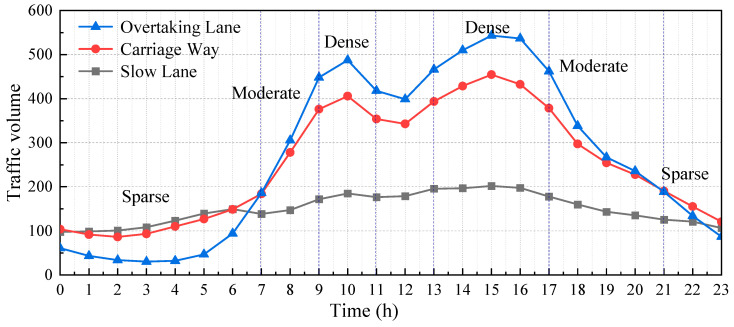
Change trend of daily average hourly traffic volume.

**Figure 17:**
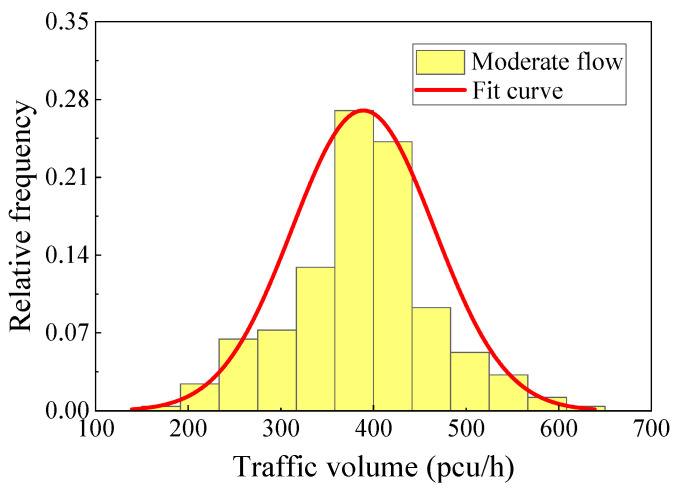
Traffic volume (moderate flow).

**Figure 18:**
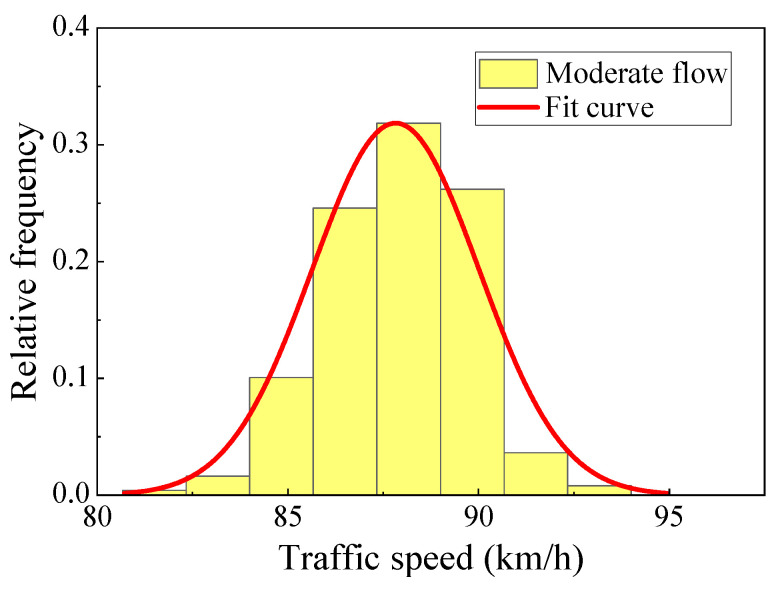
Vehicle flow speed (moderate flow).

The traffic data collected by a weigh-in-motion system (WIM) included the vehicle data of the region from 1 March to 31 March 2015, including the vehicle wheelbase, axle load, vehicle speed, and other details [27]. Figure 16 shows the hourly traffic volume results obtained from the collected data by time and lane statistics. The daily traffic volume changed greatly. On the basis of the action area of the traffic flow with obvious changes in traffic volume, the traffic flow level was divided into three intensity levels: dense, moderate, and sparse. The traffic flow intensive periods were mainly concentrated at 9:00–11:00 and 13:00–17:00, and the sparse flow period was from 21:00 to 7:00. The vehicle traffic characteristics at different intensity levels were fitted to obtain the distribution characteristics of the traffic volume and vehicle flow speed under the three intensity levels, as shown in Figure 17 and Figure 18.

**Figure 19:**
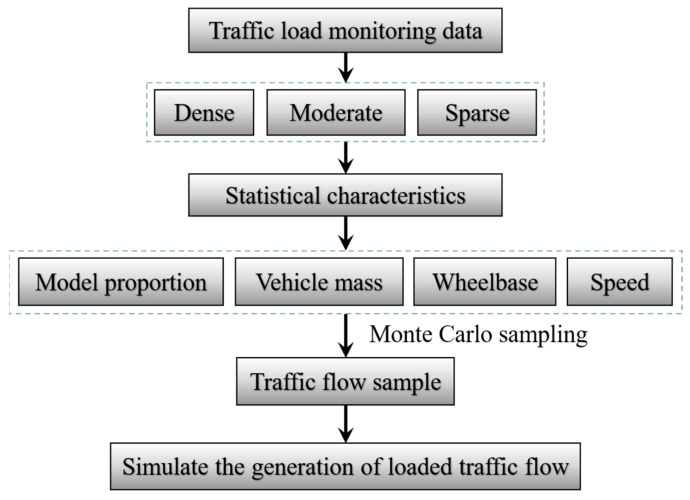
Vehicle flow simulation.

Based on the statistical results of traffic volume and traffic flow speed, the average spacing of vehicles could be determined according to Equation (14). The key parameters of traffic flow are given in the Table 7, and the characteristics of traffic flow parameters could then be determined. In combination with the random traffic flow sampling method based on the Monte Carlo method, vehicle samples were obtained [28], so as to select the traffic flow in typical representative periods for a load effect analysis. The specific traffic flow simulation process is shown in Figure 19.

(14) Q=K·V

where *Q* denotes traffic volume, *K* denotes traffic density, and *V* denotes traffic speed.

### 5.3. Numerical Analysis

Based on the above vehicle flow load simulation method, the dense, moderate, and sparse evacuation flows obtained from the sampling were loaded into the bridge finite element model. The vehicle bridge coupling analysis system established on the basis of finite element analysis software ANSYS in a previous study was used to obtain the bridge suspender response. For suspension bridges, short suspenders in the middle of the span are the most easily damaged suspenders, because of their short length and the large bending stress caused by the relative displacement between the main cable and the main girder. As the link element cannot directly give the bending stress, Wyatt’s theoretical formula was introduced to calculate the bending stress, according to the axial stress of the suspender and the angle generated by the relative movement between the main cable and the stiffening girder [29].

(15) $$\sigma_{b}=\tan\theta \cdot \sqrt{\sigma_{a}E}$$

where $\sigma_{a}$ is axial stress, *E* is elasticity modulus of steel wire, θ is the angle caused by the relative movement between the main cable and the stiffening girder.

**Figure 20:**
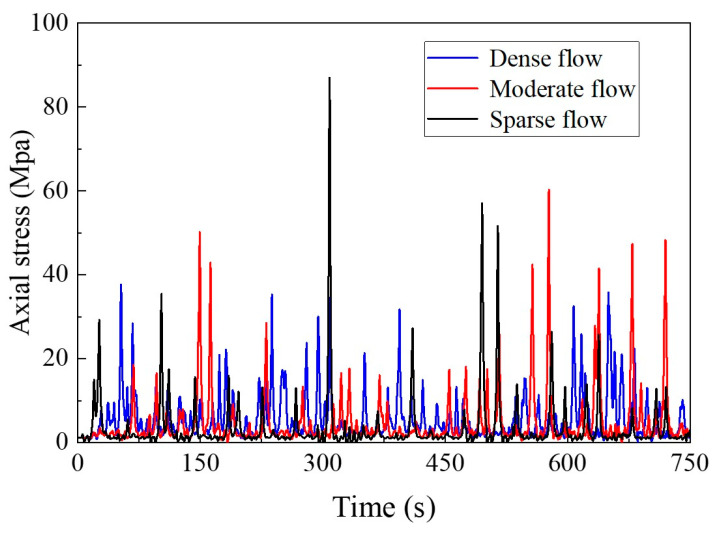
Axial stress response.

**Figure 21:**
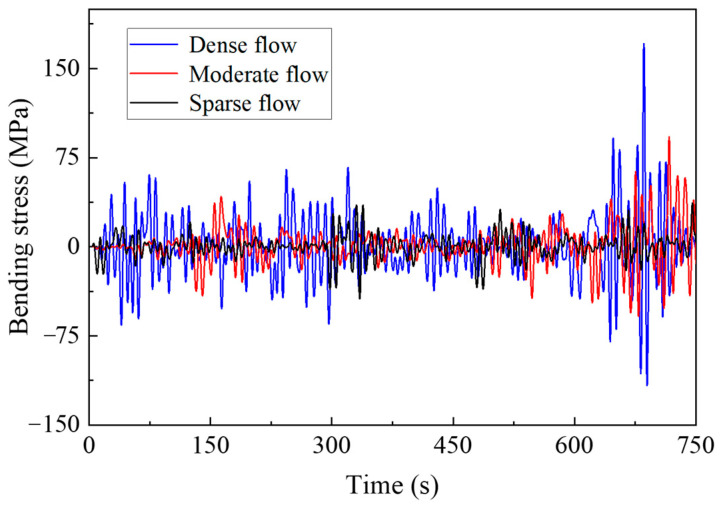
Bending stress response.

Figure 20 and Figure 21 show the stress response results of short suspenders in the mid span. The response of the suspenders under the overall traffic flow is affected by the number of vehicles and the distance between vehicles, and the response under a dense flow is most obvious. However, during driving, a driver spontaneously maintains a safe distance, to ensure safety requirements, which is usually greater than the distance between adjacent suspenders. Therefore, the extreme value of the axial force response of suspenders under sparse or moderate flow may exceed the extreme value of axial force under a dense flow, owing to the impact of single vehicle weight at the time of traffic flow. The bending stress is mainly affected by the continuous superposition of traffic flow effects, and the overall response level and extreme value of bending stress under a dense flow are the most obvious.

**Figure 22:**
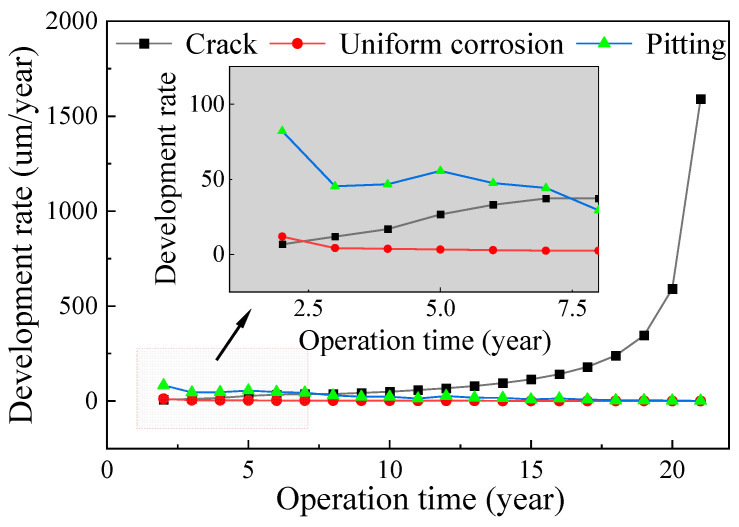
Corrosion rate of galvanized steel wire.

**Figure 23:**
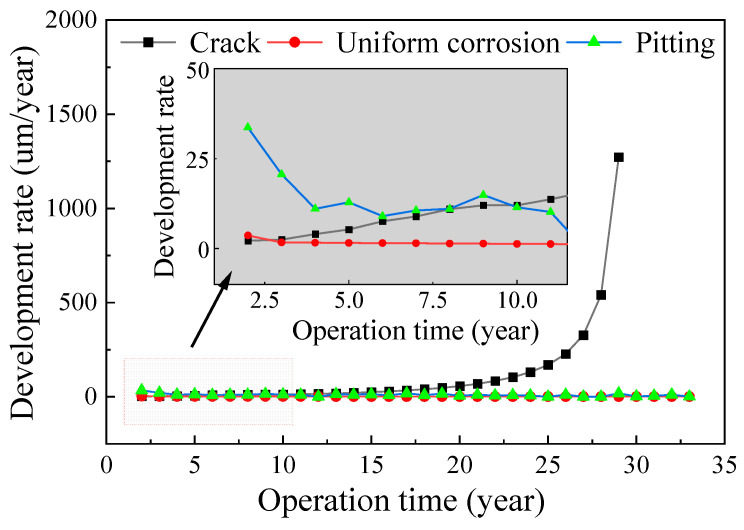
Corrosion rate of Galfan steel wire.

Basing on the obtained stress responses of suspender steel wires under different traffic flows, and combined with the uniform corrosion and pitting corrosion laws of steel wires obtained from the tests, the corrosion fatigue degradation of steel wires was analyzed. Based on the proportion of traffic flow at different levels determined from the traffic flow monitoring data, the corrosion fatigue of the two types of steel wire under the combined action of traffic flow was simulated by sampling. Figure 22 and Figure 23 show the change rules of the uniform corrosion rate, pitting rate, and crack development rate of steel wire. The initial rate of uniform corrosion and pitting corrosion was fast and then rapidly decreased. On the contrary, the crack growth rate gradually increased, and the rate increased with time. The crack growth rate of galvanized steel wire exceeded the corrosion rate after seven years of corrosion, whereas that of galvanized aluminum steel wire exceeded the corrosion rate 10 years later. Although the time difference was small, the corrosion rate of galvanized aluminum steel wire was lower, the relative crack growth rate was also significantly lower than that of galvanized steel wire, which is conducive to improving the service life of steel wire.

**Figure 24:**
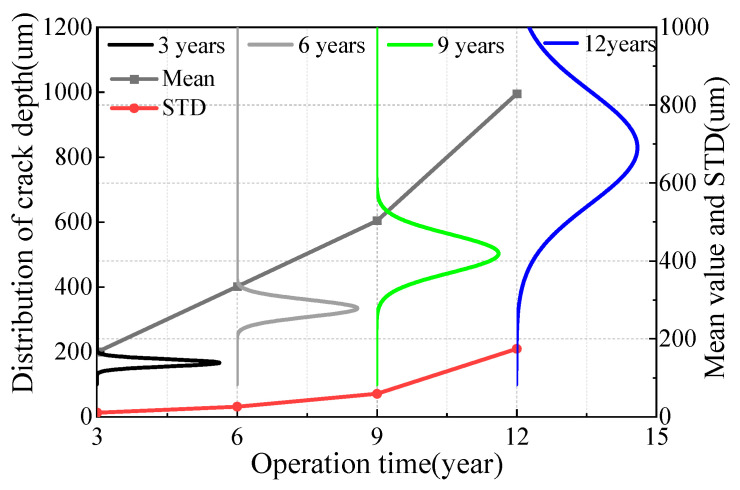
Crack depth distribution of galvanized suspender steel wire.

**Figure 25:**
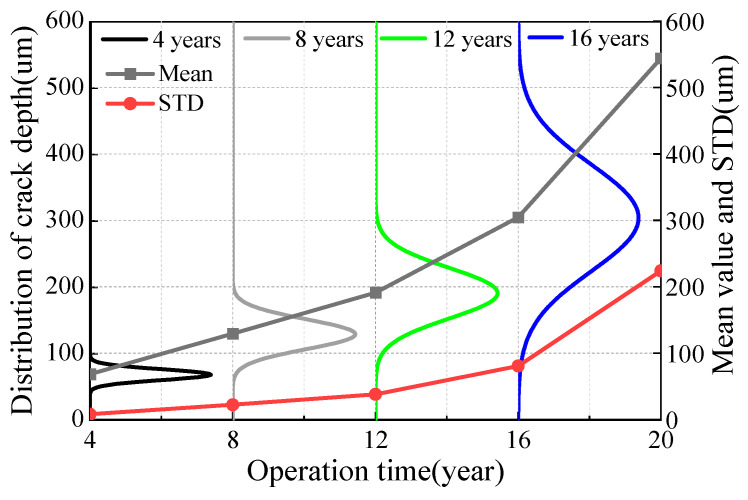
Crack depth distribution of Galfan suspender steel wire.

Figure 24 and Figure 25 show the distribution law of steel wire crack depth at different stages. With the increase of service time, the average crack depth of the whole steel wire increased and the development rate increased, which is consistent with the analysis results in the above figure. However, owing to the randomness of the pitting corrosion and crack development, the STD value of crack distribution was large at the end of service, and the discreteness of wire life became obvious.

**Figure 26:**
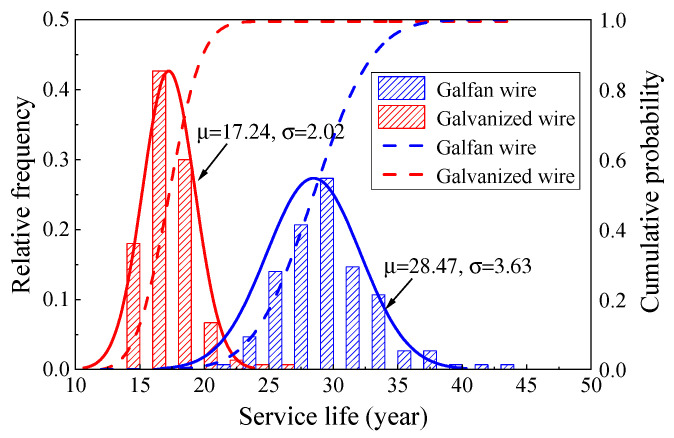
Life distribution of steel wire under comprehensive traffic flow.

**Figure 27:**
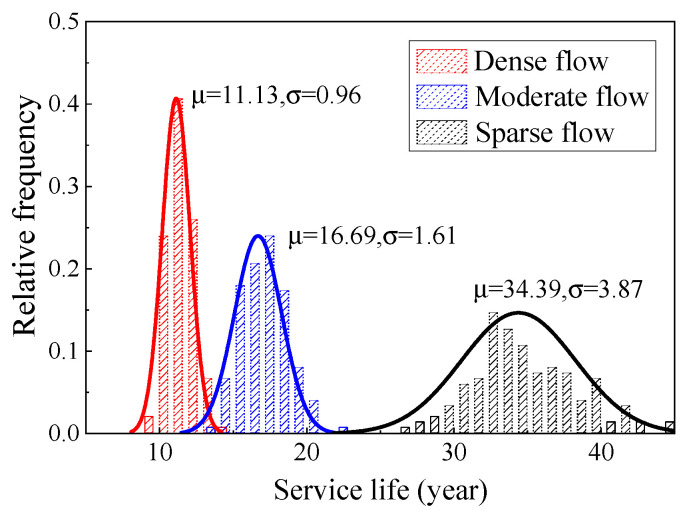
Service life of galvanized suspender wire.

**Figure 28:**
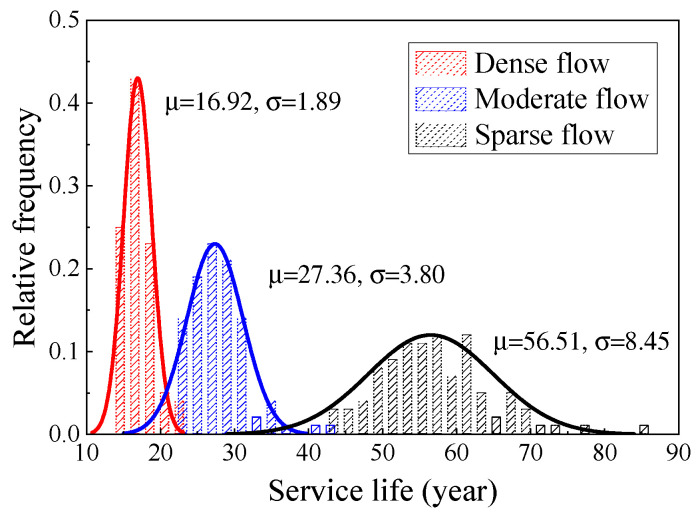
Service life of Galfan suspender steel wire.

Figure 26 shows the life distribution of the two types of steel wire under a comprehensive traffic flow. The average life of Galfan steel wire was significantly higher than that of the galvanized steel wire, being 28.47 years and 17.24 years, respectively. Figure 27 and Figure 28 show the corrosion fatigue life of the two kinds of coated steel wires under different strengths of traffic flow. The strength level of the traffic flow was the decisive factor affecting the steel wire degradation. The durability of Galfan steel wire was obviously better than that of galvanized steel wire, and the overall life of the steel wire was longer. Nevertheless, the average life of galvanized steel wire was 11.13 years, whereas that of the Galfan steel wire was 16.92 years under dense traffic flows. The corrosion fatigue life of steel wire was not significantly increased. Under a sparse traffic flow, the service life of steel wire was better than expected and was significantly higher than the design life of general cable structures (25 years). Under rarefaction flow, the average life of the two kinds of steel wires could reach 35 years and 55 years, respectively. When the proportion of sparse traffic flow and moderate traffic flow is relatively high during bridge operation, the slow corrosion fatigue degradation of Galfan steel wire at this stage could greatly improve the service life of steel wire. However, when the traffic flow intensity level is generally high, obtaining good results using Galfan steel wire would be difficult.

## 6. Conclusions

This paper took the difference in the corrosion fatigue degradation characteristics of galvanized and Galfan high-strength steel wire coatings as the research goal. It analyzed the difference of uniform corrosion and pitting corrosion of two coatings under the same conditions through an accelerated corrosion test. It then constructed a dynamic distribution model of uniform corrosion and pitting corrosion during coating corrosion. On the basis of an accelerated corrosion test and the traffic load monitoring data of a large bridge, the corrosion fatigue degradation of two kinds of steel wires under traffic load during operation was investigated. The following conclusions were obtained:

(1) The macro morphology of the corrosion of high-strength steel wire had obvious stage change characteristics. At the early stage of corrosion, the steel wire coating could effectively protect the iron matrix, and the corrosion products were free of Fe oxides. When the corrosion developed to a certain stage, brown corrosion products began to appear; on the galvanized steel wire at 386 h and on the Galfan steel wire at 1016 h. The surface coating began to be consumed, and the steel wire Fe matrix started to corrode. At this time, the steel wire surface a reddish brown rust appeared. The corrosion of steel wire can be generally divided into two parts. The first part is the corrosion of the surface coating. When the corrosion depth exceeds the coating thickness, corrosion of the second part of the steel wire matrix starts. After the corrosion products are removed, uneven pits appeared on the steel wire surface. The corrosion resistance of Galfan steel wire is obviously better than that of galvanized steel wire.
(2) The development law of steel wire corrosion depth was fitted and analyzed. In addition, a uniform corrosion development model and pitting corrosion probability model of galvanized and Galfan steel wire were established. With the extension of corrosion time, the uniform corrosion depth of zinc coating and Galfan coating conformed to the exponential increase trend. The development trend of the two coatings was similar, and the corrosion rate gradually slowed down with the increase of time. The corrosion coefficient of galvanized steel wire conformed to normal random distribution, whereas that of Galfan steel wire conformed to Cauchy distribution. The section distribution of the maximum pitting coefficient did not reject the Gumbel distribution. The location and scale parameters of the maximum pitting coefficient distribution in the two coating intervals showed an exponential downward trend with the increase of corrosion duration.
(3) The early rate of uniform corrosion and pitting corrosion was fast, and then it decreased rapidly, whereas the crack growth rate gradually increased. With the increase of time, the rate increased. The average value of the overall crack depth of the steel wire also increased, and the development rate continued to increase. The STD value of the crack distribution in the late service period was large, and the discreteness of the steel wire life became obvious. Based on the analysis of the corrosion fatigue history of steel wire under different strength traffic loads, the corrosion characteristics of steel wire was the main factor affecting its service life when the traffic strength level was low. The average service life of Galfan steel wire was significantly higher than that of galvanized steel wire. However, under a dense traffic flow with high strength, the service life of steel wire was mainly controlled by the traffic load, and the service life of Galfan steel wire increased slightly. Effective anti-corrosion measures are the key to improving the service life of steel wire.

It should be mentioned that the corrosion fatigue properties of coated steel wires were investigated through accelerated corrosion tests. However, the period of the accelerated corrosion test was relatively short, which is quite different from the real environment in service. In a subsequent study, the corrosion rate and characteristics of the steel wire need to be investigated using field exposure tests and cable components in service, which could better determine the corrosion characteristics and effects of traffic load on the corrosion performance of steel wire coatings.

## Figures and Tables

**Table 1:** Accelerated corrosion period of high-strength steel wires.

Galvanized Steel Wire		Galfan Steel Wire			
Corrosion Duration (h)	Amount of Test Pieces	Corrosion Duration (h)	Amount of Test Pieces	Corrosion Duration (h)	Amount of Test Pieces
24	5	24	5	606	10
48	5	48	5	686	10
96	5	96	5	784	10
168	5	168	5	848	10
216	5	216	5	944	10
264	5	264	5	1016	10
336	5	336	5	1088	10
384	5	384	5	1192	10
438	5	438	5	1389	10
510	5	510	5	1445	10

**Table 2:** Steel wire sample parameters.

Type of Steel Wire	Diameter (mm)	Out of Roundness	Tensile Strength (MPa)	Yield Strength (MPa)	Elastic Modulus (MPa)	Coating Quality (g/m^2^)
Galvanized steel wire	5.34	0	1895	1760	2.08 × 10^5^	336
Galfan steel wire	5.25	0	1926	1775	2.08 × 10^5^	337

**Table 3:** Mass percentages of microelements of the high-strength steel wires coating (%).

Material Composition	Zn	Al	Si	Mn	P	S	Cr
Galvanized Coating	≥99	/	/	/	/	/	/
Galfan Coating	≤95	≤5	0.24	0.86	0.009	0.002	0.17

**Table 4:** Instruments and chemical reagents.

Instrument Name	Electronic Balance	C_2_H_5_OH	NaCl	Pickling Agent
Parameter grade	0.0001 g	Analytically pure	Analytically pure	Analytically pure

**Table 5:** Technical parameters of the salt spray box.

Internal Dimensions (mm)	Test Room Temperature (°C)	Pressure Barrel Temperature (°C)	Compressed Air Force (kgf/cm^2^)
1200 × 1000 × 500	NSS ACSS 35 ± 1 CASS 50 ± 1	NSS ACSS 47 ± 1 CASS 63 ± 1	1.00 ± 0.01

**Table 6:** Mass loss of the cold-rolled carbon steel sheet.

Specimen Number	Specimen 1	Specimen 2	Specimen 3	Specimen 4
Mass loss (g/m^2^)	75	78	77	80

**Table 7:** Key parameters of traffic flow.

Key Parameter		Passing Lane	Carriage-Way	Slow Lane
Dense flow	Traffic volume (pcu/h)	572	474	209
	Traffic speed (km/h)	93.36	88.4	70.76
Moderate flow	Traffic volume (pcu/h)	451	378	184
	Traffic speed (km/h)	94.12	87.48	67.98
Sparse flow	Traffic volume (pcu/h)	175	186	132
	Traffic speed (km/h)	92.12	81.80	67.98

## Data Availability

Some or all data, models, or codes that support the findings of this study are available from the corresponding author upon reasonable request.
